# Gut microbiota facilitate adaptation of invasive moths to new host plants

**DOI:** 10.1093/ismejo/wrae031

**Published:** 2024-02-29

**Authors:** Shouke Zhang, Feng Song, Jie Wang, Xiayu Li, Yuxin Zhang, Wenwu Zhou, Letian Xu

**Affiliations:** State Key Laboratory of Subtropical Silviculture, Zhejiang A&F University, Hangzhou 311300, China; State Key Laboratory of Subtropical Silviculture, Zhejiang A&F University, Hangzhou 311300, China; State Key Laboratory of Subtropical Silviculture, Zhejiang A&F University, Hangzhou 311300, China; State Key Laboratory of Biocatalysis and Enzyme Engineering, School of Life Sciences, Hubei University, Wuhan 430062, China; State Key Laboratory of Biocatalysis and Enzyme Engineering, School of Life Sciences, Hubei University, Wuhan 430062, China; Ministry of Agricultural and Rural Affairs Key Laboratory of Molecular Biology of Crop Pathogens and Insect Pests & Key Laboratory of Biology of Crop Pathogens and Insects of Zhejiang Province, Institute of Insect Science, Zhejiang University, Hangzhou 310058, China; State Key Laboratory of Biocatalysis and Enzyme Engineering, School of Life Sciences, Hubei University, Wuhan 430062, China

**Keywords:** gut microbiome, pioneer population, Hyphantria cunea, genome resequencing, unsuitable hosts

## Abstract

Gut microbiota are important in the adaptation of phytophagous insects to their plant hosts. However, the interaction between gut microbiomes and pioneering populations of invasive insects during their adaptation to new hosts, particularly in the initial phases of invasion, has been less studied. We studied the contribution of the gut microbiome to host adaptation in the globally recognized invasive pest, *Hyphantria cunea*, as it expands its range into southern China. The southern population of *H. cunea* shows effective adaptation to *Metasequoia glyptostroboides* and exhibits greater larval survival on *Metasequoia* than the original population. Genome resequencing revealed no significant differences in functions related to host adaptation between the two populations. The compatibility between southern *H. cunea* populations and *M. glyptostroboides* revealed a correlation between the abundance of several gut bacteria genera (*Bacteroides*, *Blautia*, and *Coprococcus*) and *H. cunea* survival. Transplanting the larval gut microbiome from southern to northern populations enhanced the adaptability of the latter to the previously unsuitable plant *M. glyptostroboides*. This research provides evidence that the gut microbiome of pioneering populations can enhance the compatibility of invasive pests to new hosts and enable more rapid adaptation to new habitats.

## Introduction

Herbivorous insects can be influenced by various biotic or abiotic factors, such as climate change and human activities, and can undergo active or passive dispersal [1-4]. Insects often establish and proliferate locally through their strong adaptability in new environments [1, 5-7]. Some insects are invasive (non-native species causing ecological or economic harm) and can have a significant impact on both the environment and the human economy [5, 8]. An important question is how invasive species are able to rapidly establish and adapt to new environments [6, 9, 10]. A factor contributing to successful colonization and invasion is genomic evolution and rapid environmental selection [11-14]. However, the evolution and stabilization of adaptive traits at the genomic level pose considerable challenges and often require a timescale of thousands, or millions, of years [15-17]. Thus, invasive insects face difficulties in achieving rapid genomic adaptation in new environments within a short timeframe [18-20]. It is not surprising that these agents can also be involved in biological invasions and host adaptation given the widespread role of insect gut microbiota in various life activities [5, 21-23]. For example, alterations in intestinal microbe composition in the oriental fruit fly (*Bactrocera dorsalis*) can influence metabolism and transcriptional regulation and promote fly development [23]. Similar outcomes have been observed in other invasive insects, showcasing their ability to enhance development, facilitate intraspecies communication, and detoxify plant defensive chemicals [2-4]. These functionalities aid invasive insects by improving their adaptability; however, it is unclear whether these functions directly determine the success of invasive insects in adapting to new environments. There is little direct evidence regarding whether, and how, gut microbiota assist invasive species, particularly pioneer species (the initial colonizers of newly invaded environments) [24], in rapidly adapting to new environments.

The fall webworm*, Hyphantria cunea*, is one of the most invasive agricultural and forest pests worldwide [6, 9]. Since its accidental introduction to northeast China in 1979, *H. cunea* has spread to 13 provinces across the country [25]. Monitoring has now confirmed the migration of *H. cunea* across the Yangtze River into the southern subtropical regions of China (e.g. Zhejiang province) [6, 9, 26]. Gymnosperms, including *Metasequoia glyptostroboides* and *Taxodium distichum*, now serve as acceptable host plants for *H. cunea* populations in southern China, in contrast to the host plants used by northern populations [26, 27]. Studies on *H. cunea* populations in the southern subtropical region of China show a preference for oviposition on the dominant plants within the geographical range of the invasion [26]. Also, the oviposition preferences of the southern *H. cunea* populations on *Populus simonii* and *M. glyptostroboides* differ from those of northern *H. cunea* populations (e.g. Beijing population) [26], suggesting a rapid adaptation of *H. cunea* to the newly invaded environment. Exploring the adaptive mechanisms of the southern subtropical *H. cunea* population hosts is crucial for the management of the *H. cunea* southern expansion pioneer population, as well as for understanding its potential outbreak mechanisms.

*H. cunea* in China has undergone a significant expansion of gene families related to carbohydrate metabolism and taste receptors, coupled with the presence of notable functional polymorphisms [6, 10]. These observations suggest that *H. cunea* possesses adaptive taste ability and the capacity to efficiently utilize novel nutrients from new hosts [6, 28]. Additionally, the complex microbiome in the *H. cunea* gut plays an important role in *H. cunea* host adaptation [28, 29]. For example, *Hydrogenophaga* and *Acinetobacter* in *H. cunea* can contribute to the breakdown of cinnamic acid, a defensive secondary metabolite in plants [28]. In response to plant secondary metabolites from different host plants, the gut microbiome of some herbivorous insects may exhibit significant changes in its structure and function. This could potentially lead to an enrichment of the core microbiota that are particularly adept at degrading plant secondary metabolites and possibly aid the host insect in tolerating phytochemical resistance [28, 30]. Although it has been established that host plants can induce variations in the composition and function of *H. cunea* bacterial flora [29, 31], two scientific questions arise in the context of *H. cunea*’s southern expansion: (1) Is the altered gut microbiome in *H. cunea* pioneer populations correlated with host plant compatibility in the new habitat? and (2) to what extent does the gut microbiome influence the acclimatization of *H. cunea* to new host plant species?

To address these questions, we initially assessed the compatibility of larvae from the southern *H. cunea* population with various host plants within the invaded southern ranges. We used high-throughput sequencing technologies to characterize the microbiome of the *H. cunea* larvae obtained from different compatible host plants by sequencing 16S rRNA full-length amplicons, Internal transcribed spacer (ITS) full-length amplicons, and metagenomic sequences. We then identified microorganisms within the microbiomes that were significantly associated with the survival rates of *H. cunea*. The role of gut microorganisms in synergistically adapting to new hosts and tolerating diverse host chemical resistances was compared and analyzed through population genome resequencing of the *H. cunea* from Beijing and Zhejiang populations, as well as via the elimination and re-introduction of gut microorganisms. This study provides insights into the compatibility of pioneer populations of invasive insects with local host plants, and it illustrates the significant role of gut microorganisms in facilitating rapid adaptation of invasive pests to new environments.

## Materials and methods

### Test material

The subtropical *H. cunea* pioneer population (Zhejiang *H. cunea* population) was collected from Jiashan County (30°51′14″N, 120°54′36″E), Jiaxing City, Zhejiang Province, in July 2020. The Zhejiang *H. cunea* population and Beijing *H. cunea* population (Provided by the Chinese Academy of Forestry) were cultured using artificial diet [wheat germ (12%), soybean powder (12%), sucrose (1%), ascorbic acid (0.5%), choline chloride (0.5%), vitamin B complex (0.03%), sorbate (0.15%), anhydrous citric acid (0.5%), corn oil (0.3%), carrageenan powder (2.5%), AGAR powder (6.4 g), and water (320 ml)] provided by the Chinese Academy of Forestry in a controlled environment with a relative humidity range of 70%–80%, 16:8 h (L:D) photoperiod, and a temperature of 25 ± 1°C. For testing purposes, fourth instar larvae of uniform size were chosen. Two-year-old saplings from five host plants, namely *Morus alba*, *Carya illinoinensis*, *Carya cathayensis*, *P. simonii*, and *Metasequoia glyptostroboides*, were selected for testing at the orchard test base of Zhejiang A & F University (30°12′33″N, 119°21′14″E). The saplings were cultivated in greenhouses.

### Determination of compatibility of Zhejiang *H. cunea* larvae on different hosts

Two-year-old potted seedlings with consistent growth status were selected from saplings of each plant species. The top young shoots and lower aged leaves were trimmed to standardize the sample. The plants were then acclimatized in a cage for 3 days after their wounds had healed. Both the saplings and larvae enclosures were sterilized with 75% alcohol. After 3 days of exposure to air, allowing the alcohol to completely evaporate, each sapling species was individually inoculated with Zhejiang *H. cunea* larvae (*n* = 150, 30 larvae/replicate) that had been starved for 24 h [32-34] and had emptied their guts beforehand. Simultaneously, the control group of larvae (*n* = 150, 30 larvae/replicate) was provided with an artificial diet. Daily observations of the larval growth were conducted, and the mortality rate was recorded over a 15-day observation period. On Day 15, the number and weight of the surviving larvae were recorded. To highlight the adaptation results of the Zhejiang *H. cunea* population, the Beijing *H. cunea* larvae were also processed in the same way, and mortality data were collected. The survival rate of larvae was analyzed using GraphPad Prism 9 software by conducting the log-rank test and drawing a survival curve. The comparison of the number of surviving larvae and the weight of surviving larvae among different treatment groups was conducted using one-way Analysis of variance (ANOVA) with a Bonferroni post hoc test.

### Extraction of Zhejiang *H. cunea* larval gut microbial DNA and analysis of 16S rRNA gene and ITS gene data

After 15 days, the surviving Zhejiang *H. cunea* larvae were collected from each sapling species. Following a 24-h starvation period, the larvae were dissected in a sterile Phosphate buffered saline (PBS) buffer solution, and their guts were opened using sterile scissors. The gut samples were placed in a sterile Plain end (PE) tube containing 2 ml of PBS buffer, sealed, and then shaken for 1 min in an ultrasonic shaker. Three guts were grouped together as one sample. In total, 30 samples were obtained from the five host plant species and one artificial diet treatments and subsequently stored at −80°C.

The total DNA of the gut microbiome was extracted using the QIAGEN DNeasy Blood & Tissue Kit (QIAGEN, USA). Gut samples were stored at −80°C and then thawed at 4°C before being homogenized using an aseptic homogenizer. The homogenized gut solution was centrifuged at 7500 rpm, and the resulting supernatant was transferred to a new sterile PE tube for total DNA extraction. After quantifying the purity and concentration of the DNA samples, we selected the full-length 16S rRNA gene (~1500 bp) and full-length ITS gene (~1300 bp) for sequencing. To achieve this, we conducted PCR amplification using 16S rRNA full-length specific primers (forward 27F AGAGTTTGATCMTGGCTCAG, reverse 1492R ACCTTGTTACGACTT) and ITS full-length specific primers (forward ITS1F CTTGGTCATTTAGAGGAAGTAA, reverse LR3 CCGTGTTTCAAGACGGG). The target fragments were amplified with 2% agarose gel electrophoresis and then recovered using the Axygen gel recovery kit (Axygen, Shanghai, China). A quantitative assay of Polymerase chain reaction (PCR) products was conducted on a microplate reader (BioTek, FLx800, USA) utilizing the Quant-iT PicoGreen dsDNA Assay Kit (Calbiochem, 1 ml kit, DEU). After the samples were qualified, they were mixed according to the required data amount for each sample. The Illumina TruSeq Nano DNA LT Library Prep Kit (Illumina, FC-121-4002, USA) was used for constructing the library with kit-specific steps. Prior to on-board sequencing, the Agilent High Sensitivity DNA Kit (Agilent Technologies, 5067-4626, USA) was used to evaluate libraries using the Agilent Bioanalyzer. Verified libraries were subsequently subjected to double-ended sequencing utilizing the NovaSeq 6000 SP Reagent Kit (Illumina, v1.5, USA) on the NovaSeq System (Illumina, NovaSeq 6000, USA).

QIIME2 (version 2019.4) was utilized to decode the original sequence data employing the Demux plug-in and the Cutadapt plug-in for primer excision [35]. The sequences were subjected to quality filtering, de-noising, splicing, and chimera removal with the DADA2 plug-in [36]. Characteristic sequence Amplicon sequence variants (ASVs) were created by merging sequences with 100% sequence similarity. The taxonomic information corresponding to each ASV was obtained by comparing the obtained bacterial and fungal ASV characteristic sequences with reference sequences in the Greengenes2 database [37] and UNITE (https://unite.ut.ee/) [38] databases. We flattened the bacterial and fungal data using the feature table rarefy function in QIIME2 [35]. The rarefying depth was set to 95% of the minimum sample sequence size to obtain the normalized abundance data table for subsequent analysis. The Good Coverage, Shannon, and Observed Species indices of the alpha diversity were computed for all of the bacteria and fungi samples utilizing the QIIME2 software. The “qiime diversity core-metrics” command was invoked to compute the Bray–Curtis distance matrix based on the sample Bray_curtis distance matrix. The resultant distance matrix was then subjected to PCoA and a permutational multivariate analysis of variance consistent with a permutation test by using the vegan package in R software.

Using the modular analysis method as previously described [39], six groups of bacteria and fungi were analyzed. Modularity refers to the extent to which a network is organized into distinct modules (ecological clusters) [40, 41]. These modules are characterized by a higher concentration of connections within themselves compared to the connections between different groups. Spearman correlation analysis was conducted utilizing the vegan package of R software to calculate the correlation between ASV relative abundance and surviving number of larvae. A correlation coefficient with an absolute value greater than 0.6 and *P* < .05 was considered significant. After importing the data table into the Gephi software and conducting module analysis [39], we obtained the top eight module genus abundance files. Then, we correlated genus abundance from different modules with the larval survival rate and analyzed the significance of the correlation fit. Species difference analysis was conducted at the genus level in the modules showing significant positive and negative correlations with the number of surviving larvae. R software was used for analysis, and the clustering heat map was used to display the identified different species. Subsequently, ASV enrichment in each module was determined and specific metabolic pathways were mined from the MetaCyc database.

### Evaluating the compatibility of antibiotic-treated or untreated Zhejiang *H. cunea* larvae on *M. glyptostroboides*, with metagenomic analysis of the gut microbiome in both groups

Two-year-old potted *M. glyptostroboides* seedlings at the same growth stage were chosen. The upper young branches and lower old leaves were pruned, followed by rinsing with 75% alcohol and air-drying. The leaves of the treatment group were immersed in sterile water with antibiotics [tetracycline (0.025%), cephalexin (0.025%), rifampicin (0.025%), and gentamicin (0.025%)], and the control group was placed in sterile water. The treatments were conducted for 30 min, and the leaves were then placed in sterile Petri dishes to air dry. The fourth instar Zhejiang *H. cunea* larvae (*n* = 200) were subsequently assigned to two groups. After 24 h of food deprivation to empty the gut cavity, the larvae were randomly placed in sterile Petri dishes. One group of larvae (*n* = 100) fed on *M. glyptostroboides* leaves treated with antibiotics. The other group of larvae (*n* = 100) ate *M. glyptostroboides* leaves treated with water. Larval growth was observed daily, and mortality was recorded for 15 consecutive days. The entire experiment was repeated eight times. The survival rate, larval weight, and the number of survivors were subjected to the log rank test using GraphPad Prism 9 software, and a survival curve was plotted. The difference in larval body weight between the two groups was examined using an independent *t*-test.

From the surviving larvae, 90 larvae were randomly selected from each group for further analysis. Sterile scissors were used to cut each larvae gut, and three guts were placed in a centrifuge tube filled with 2 ml of sterile PBS buffer. After sealing, the tube was put in an ultrasonic shaker for 1 min. A total of 30 samples were stored at −80°C for testing. Whole genome shotgun sequencing was performed on the metagenomic DNA extracted from the samples. DNA was sequenced using the 150 bp paired end libraries on the Novaseq and Hiseq HTS Systems (Illumina), resulting in an average output of 10 GB per sample. The demultiplexed metagenome raw data can be found in the BIG Submission (PRJCA022940). The data were filtered for quality using Cutadapt (v1.17) to obtain clean sequence data. Species annotation was performed using Kraken2, and assembly was carried out with the Megahit software, retaining only contigs above 200 bp. The available ASV annotation data for the contig sequences were integrated with abundance tables for every sample to determine the overall species abundances at every taxonomic rank (domain, phylum, class, order, family, and genus) within each community. The MetaGeneMark program, accessible through http://exon.gatech.edu/GeneMark/, was used to identify open reading frames, predict coding sequences, and acquire protein annotations. The non-redundant protein sequences were subsequently compared to the Kyoto Encyclopedia of Genes and Genomes (KEGG) databases to further annotate gene functions for the samples [42]. OmicShare online software was used for analyzing the abundance of KEGG functions in communities, with ggTree and other R packages being used to present these outcomes. To enhance comprehension of the pathways of plant secondary metabolite degradation within *H. cunea* gut microbiomes, genomic binning assembly was performed for the metagenomic data on microbial strains. To clarify the role of the top 20 KEGG pathways in plant secondary metabolite degradation, we annotated microbe genomes from the binning analysis against the top 20 KEGG pathways. The correlations were analyzed in the data and displayed using a Sankey diagram.

### Determining the role of gut microbiota and genomic SNP in the differential compatibility of Zhejiang and Beijing *H. cunea* larvae on *M. glyptostroboides*

To ascertain whether gut microorganisms influence the differential responses of *H. cunea* larvae populations to *M. glyptostroboides*, we conducted feeding experiments using axenic larvae from both Beijing and Zhejiang *H. cunea*, with conventionally reared Zhejiang *H. cunea* serving as controls. For acquisition and maintenance of axenic larvae, newly emerged *H. cunea* larvae were promptly subjected to a sterile environment and sustained by feeding an artificial diet containing antibiotics [tetracycline (25 mg/g), cephalexin (25 mg/g), rifampicin (25 mg/g), gentamicin (25 mg/g)]. Gut sterility was assessed through direct bacterial plating on Luria-Bertani (LB) agar and Realtime fluorescence quantitative PCR (qPCR) assays. Axenic and conventional fourth instar larvae (*n* = 6) were randomly selected, and their intestines were homogenized upon collection. The resulting homogenate was split into two equal parts, one for a plating experiment and the other for RNA extraction. Following the transcription of total RNA into cDNA, bacterial expression levels were quantified using the primers q16S-f TCCTACGGGAGGCAGCAGT and q16S-r GGACTACCAGGGTATCTAATCCTGTT (Fig. S5). Then, the fourth instar Zhejiang *H. cunea* (*n* = 300), axenic Zhejiang *H. cunea* (*n* = 300), and axenic Beijing *H. cunea* (*n* = 300) were fed on *M. glyptostroboides* leaves. Larval growth was observed daily, and mortality was recorded for 15 consecutive days. The larval survival rate was analyzed with GraphPad Prism 9 software by conducting a log-rank test and drawing the survival curve. Comparison of the number of surviving larvae and the weight of surviving larvae among different treatment groups was conducted using one-way ANOVA with a Bonferroni post hoc test.

For comparing the genomic differences between the Zhejiang *H. cunea* population and the Beijing *H. cunea* population, we performed genome resequencing. Twenty *H. cunea* fourth instar larvae from Zhejiang and Beijing populations were randomly selected, and their DNA was extracted using the aforementioned method. Double-terminal sequencing of these libraries was performed using high-throughput sequencing based on the NovaSeq System. The analysis commenced with the processing of raw sequencing data using fastp software to ensure its quality. The high-quality data obtained were then aligned to the *H. cunea* reference genome using the mem algorithm of BWA software (Version 0.7.12-r1039) with the default parameters. After alignment, SAM files were sorted and converted into BAM files using picard software (Version 1.107). Single nucleotide polymorphism (SNP) calling was performed using GATK software (Version 4.5.0.0), whereas SAMtools (Version 1.19) calculated the average depth and coverage of the sequenced data on the genome. VCFtools (Version 4.1) was utilized to analyze SNP density across the genome, employing a sliding window approach with a window size of 100 kb and steps of 10 kb. PCoA was conducted on differential SNPs identified in the Beijing and Zhejiang *H. cunea* genomes, which were functionally and positionally annotated using ANNOVAR software (Version 2017-07-17). For genetic diversity analysis, the populations’ command of STACKS software (Version 2.55) calculated the genetic differentiation value (*F*_ST_) from SNP data. A sliding-window approach with 100 kb windows moving in 10 kb steps quantified polymorphism levels (*θπ*). To detect regions showing strong selective sweep signals, windows with both significantly low and high *θπ* ratios (*θπ*, BJ/*θπ*, ZJ, in the 5% left and right tails) and high *F*_ST_ values (0.299) were considered, as per the empirical distribution. Finally, genes specifically enriched in the Zhejiang *H. cunea* larvae were identified and functionally annotated through KEGG analysis.

### Effect of transplanted gut microorganisms from Zhejiang *H. cunea* larvae on Beijing *H. cunea* larvae tolerance to *M. glyptostroboides*

To investigate whether the gut microbiome of the Zhejiang *H. cunea* population, residing on *M. glyptostroboides*, can increase the tolerance of Beijing *H. cunea* to *M. glyptostroboides* toxicity, we conducted a gut microbiome transfer experiment. Preparation of artificial diet containing *M. glyptostroboides*: the artificial diet containing *M. glyptostroboides* was prepared by adding 10 g of *M. glyptostroboides* dry leaf powder to 80 g of artificial diet. The Zhejiang *H. cunea* larvae were fed on *M. glyptostroboides* until they reached the fourth instar stage, after which they were starved for 24 h to empty their guts. Three guts were then dissected in sterile PBS buffer. The guts were aseptically cut and homogenized in a sterile crucible. Then, the homogenate was mixed with 2 ml of sterile PBS buffer in a sterile PE tube. We centrifuged the mixture at 4°C and preserved the supernatant for future use.

To demonstrate the successful colonization of the transferred bacteria, we conducted fluorescent plasmid introduction on isolated *Escherichia coli* strains. After activation, 0.1 ml of 1 × 10^8^ CFU *E. coli* was mixed with 0.9 ml bacterial solution (gut supernatant from the Zhejiang *H. cunea* larvae) (BJ-ZJ-Trans), 0.9 ml inactivated bacterial solution (BI-IB, inactivated at 90°C for 30 min), and 0.9 ml sterile water (BI-SW). Then, each liquid was plated on nine Petri dishes containing *M. glyptostroboides*. Subsequently, a total of 300 fourth instar larvae from sterile lines of the Beijing *H. cunea* population were randomly selected and separated into three groups. The three groups were separately placed into each of the three different types of plates (BJ-ZJ-Trans, BI-IB, and BI-SW). Larval growth was observed daily, and mortality was recorded. The entire process of aseptic operation was documented for 15 consecutive days. The entire experiment was repeated three times. The survival rate of larvae was analyzed using GraphPad Prism 9 software by conducting the log-rank test and drawing the survival curve. The comparison of the number of surviving larvae and the weight of surviving larvae among different treatment groups was conducted using one-way ANOVA with a Bonferroni post hoc test.

Following 24-h starvation, the guts of the surviving larvae were emptied and dissected in sterile PBS buffer. The resulting guts were collected for metagenomic sequencing. An analysis of the gut microbiota of the Beijing *H. cunea* larvae after microbiota transfer (BJ-ZJ-Trans, *n* = 9) and the Zhejiang *H. cunea* larvae (ZJ, *n* = 9) after *M. glyptostroboides* feeding was conducted using metagenomic sequencing to compare the differences between the two groups. The differences in bacterial flora were compared using the Bray–Curtis distance matrix, and the genus-level species were plotted in a circular map. Following this, the expression information of genes enriched by the ko00361, ko00362, ko00643, and ko00627 pathways was obtained by analyzing metagenomic data (transcripts per kilobase per million mapped reads). The obtained expression data were subjected to correlation regression analysis in GraphPad 9 software to assess the relationship between relative gene expression and the number of *H. cunea* survivors.

## Results

### The compatibility of the southern Zhejiang *H. cunea* varies on different tree species

**Figure 1:**
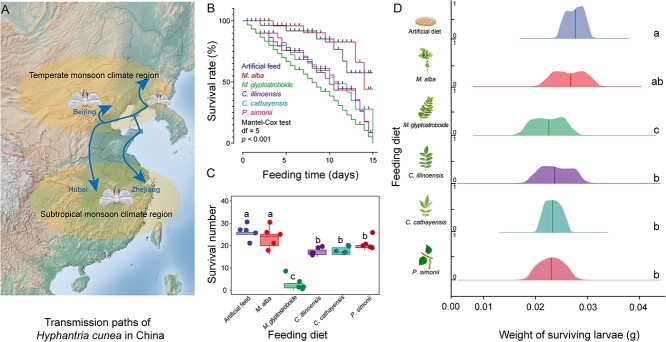
Comparison of survival rates and weights of Zhejiang *H. cunea* larvae after being fed different diets; (A) the transmission path of the *H. cunea* in China [23]; (B) larvae survival rates over a 15 days period on different diets; (C) analysis of the difference in the number of surviving larvae over 15 days on different diets; (D) analysis of the difference in larval weight over a 15 days period on different diets.

Throughout the 15-day observation period, no significant difference in survival rates of Zhejiang *H. cunea* was observed between the control group and the *M. alba* fed larvae (*P* > .05) (Fig. 1). The survival rates of these two groups were higher compared to those of the other treatment groups (*P* > .05) (Fig. 1B). No significant differences were observed in the survival rates between *P. simonii*, *C. cathayensis*, and *C. illinoinensis* (*P* > .05) (Fig. 1C). The *M. glyptostroboides* fed larvae exhibited the highest mortality rate, with only 4 ± 3 individuals surviving by Day 15 (Fig. 1C). The heaviest surviving larvae were from the control and *M. alba* groups, whereas the lightest larva was from the *M. glyptostroboides* group (Fig. 1D). The mean weights of larvae fed on *C. illinoinensis*, *C. cathayensis*, and *P. simonii* were similar (Fig. 1D).

To verify the compatibility of Zhejiang *H. cunea* with the tested trees, Beijing *H. cunea* larvae were used as a control (Fig. S1). Overall, Beijing *H. cunea* larvae exhibited a higher mortality rate compared to Zhejiang *H. cunea* larvae when exposed to the five tree species (Fig. S1). There was a significant difference in mortality rate between the two populations when fed *M. glyptostroboides* (Fig. S1, *P* < .05). Thus, the southern subtropical *H. cunea* pioneer population in Zhejiang was more compatible with *M. glyptostroboides* compared to the Beijing *H. cunea* population.

### Diets significantly influence the composition of *H. cunea* larval gut microbiome

After 15 days of feeding on the different diets, the gut microbiome diversity in *H. cunea* larvae showed significant diet-associated differences (Fig. S2). In the *M. glyptostroboides* group, the Shannon and Observed Species indices for both bacteria and fungi were significantly higher compared to those of the other groups. However, the Good Coverage index was relatively lower than that in the other groups (Fig. S2). Beta diversity analysis revealed that gut bacteria were influenced by the type of food consumed; however, no significant differences were observed in the *M. alba* and *C. illinoinensis* groups (Fig. S3A and Table S1). In contrast, significant distinctions were found among the other food groups (Fig. S3B and Table S1, *P* < .05). The tree file clustering and species composition can be categorized into five clusters (Fig. S3B). The feeding group formed a single branch based on species similarity, predominantly dominated by *Lactobacillus* (Fig. S3B). *Prevotella* was the dominant bacterium in the *M. glyptostroboides* group, which also formed a single branch (Fig. S3B). The feeding groups of *P. simonii* and *C. cathayensis* were grouped into distinct branches with primarily *Acinetobacter* and *Enterobacter*, respectively (Fig. S3B). The *C. illinoinensis* and *M. alba* groups, in contrast, were combined into a single branch with dominant species such as *Enterobacter* and *Lactobacillus* (Fig. S3B).

Feeding *H. cunea* larvae with various plant hosts had a lesser impact on the composition of gut fungal flora compared to bacterial flora. The gut fungal communities of *H. cunea* larvae fed *M. alba*, *C. cathayensis*, and *C. illinoinensis* were similar (Fig. S3C and Table S1). The fungal communities in *M. glyptostroboides* had significant differences in the *P. simonii*, *C. cathayensis*, and *M. alba* groups (Table S2, *P* < .05), suggesting that food can have some impact on the structure and composition of the fungal community. Tree file clustering revealed that the diet group and poplar group formed distinct clusters (Fig. S3D), consistent with the results of the PCoA. The *M. alba* group and *M. glyptostroboides* group showed significant branching, whereas the *C. cathayensis* and *C. illinoinensis* groups showed minor differences with mixed clusters (Fig. S3D). In the *M. alba* group samples, *Mycosphaerella* was identified as the dominant fungal group, whereas in the *M. glyptostroboides* group samples, both *Mycosphaerella* and *Aspergillus* were prevalent fungal groups. In addition, in the control samples, *Aspergillus* was identified as the dominant bacterial group (Fig. S3D).

### Presence of essential modules of the gut microbiome is associated with host plant compatibility

**Figure 2:**
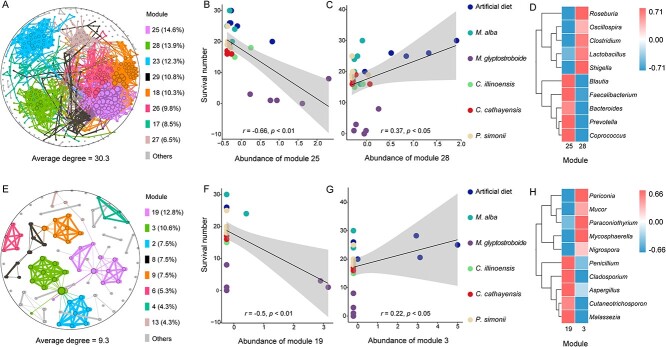
Correlation analysis of gut microbial abundance in Zhejiang *H. cunea* larvae and their survival across different diets; (A, E) network representation of bacterial and fungal communities in *H. cunea* larvae fed with artificial and five other plant-based diets; node colors indicate distinct modules, and node size corresponds to the node’s degree within the network; (B, C, F, G) linear-regression analysis correlating the relative bacterial abundance in the corresponding bacterial or fungal modules (Module 25 and 28 for bacterial communities; Module 19 and 3 for fungal communities) with the survival number of the *H. cunea*; different colors indicate different diets, including artificial feed and the other five plants; (D, H) heatmaps showing the top 10 bacterial or fungal genera with significant differences between the modules (Module 25 vs. Module 28 in bacterial communities; Module 19 vs. Module 3 in fungal communities).

To ascertain whether the structural disparities of the *H. cunea* gut microbiome (bacteria and fungi) are linked to host plant compatibility, we conducted an analysis of the correlation between microbial abundance within the microbiome modules and host plant compatibility. A correlation network diagram was constructed for microbiome bacteria when fed different host plants, resulting in the identification of eight modules with connectivity ratios exceeding 6.5% through modular analysis, and the average connectivity among ASVs was 30.325 (Fig. 2A). The fitted correlation analysis showed a negative correlation between the relative abundance of Module 25 and the survival number of *H. cunea* larvae (*r* = −0.66, *P* < .01) (Fig. 2B). Samples from this module predominantly clustered around the fitted line, indicating that module 25’s gut microbiota distribution was primarily associated with *H. cunea* larvae feeding on *M. glyptostroboides* (Fig. 2B). This bacterial module is believed to be primarily associated with tolerance to plant-resistance compounds. Also, Module 28 was positively correlated with the survival number of *H. cunea* larvae (*r* = 0.37, *P* < .05) (Fig. 2C). This suggests that the bacteria in this module are primarily associated with nutrient metabolism. When comparing the gut bacteria in the two modules, *Blautia*, *Faecalibacterium*, *Bacteroides*, *Prevotella*, and *Coprococcus* were specifically enriched in Module 25 (Fig. 2D). Also, *Roseburia*, *Oscillospira*, *Clostridium*, *Lactobacillus*, and *Shigella* were specifically enriched in Module 28 (Fig. 2D).

The analysis of fungal modules across all of the samples indicated that fungal modules showed a lower level of connectivity compared to bacterial modules, with an average connectivity degree of 9.313 between ASVs (Fig. 2E). The top eight modules accounted for over 4.2% of connectivity ratios, with Module 19 and Module 3 being the most dominant, constituting 12.8% and 10.6% of connectivity ratios, respectively (Fig. 2E). There were correlations between the relative abundance of Module 19 and Module 3 and the survival number of *H. cunea* larvae (*P* < .05) (Fig. 2F and G). Module 19 showed a negative correlation with the number of surviving larvae (*r* = −0.5). Based on the distribution of samples from the Metasequoia group around the fitting line (Fig. 2F), it appears that the fungi in this module were associated with the co-feeding of larvae on unsuitable hosts. However, Module 3 had a significant positive correlation with the number of surviving larvae (*r* = 0.22), whereas the diet samples were uniformly distributed around the fitting line (Fig. 2G). These findings suggest that the module fungus played an important role in the digestion and absorption of food nutrients. When comparing the gut fungi present in the two modules, *Penicillium*, *Cladosporium*, *Aspergillus*, *Cutaneotrichosporon*, and *Malassezia* were specifically enriched in Module 19 (Fig. 2H), which is linked to host adaptation. *Periconia*, *Mucor*, *Paraconiothyrium*, *Mycosphaerella*, and *Nigrospora* were significantly enriched in Module 3 (Fig. 2H), which is associated with nutrient metabolism.

### Analysis of key strains and their functional enrichment related to host adaptation of *H. cunea*

To identify the microbial strains crucial for *H. cunea* larvae adaptation to unsuitable hosts, we examined the relationship between ASVs in bacterial Module 25 and fungal Module 19, both associated with plant feeding and larval survival. A total of 13 ASVs were specifically enriched in the gut bacterial Module 19 and were associated with the number of surviving larvae (Fig. S4A). Two ASVs of *Bacteroides* were identified, namely B-ASV12 and B-ASV31, and both showed a negative correlation with larval survival (*r* < 0, *P* < .05) (Fig. S4A). *Blautia* and *Coprococcus* each had one retained ASV, both of which were significantly and negatively correlated with larval survival (*r* < 0, *P* < .05) (Fig. S4A). Although *Faecalibacterium* was enriched in three ASVs, no significant correlation with larvae survival was observed (*P* > .05) (Fig. S4A). Among the nine ASVs enriched by *Prevotella*, both B-ASV7 and B-ASV62 were significantly positively correlated with the number of larvae surviving (*r* > 0, *P* < .05) (Fig. S4A). Among the ASVs specifically enriched by gut fungal Module 19 in larvae, 13 ASVs were associated with the number of larvae surviving. Nine ASVs were retained in *Aspergillus*, all showing a negative, but nonsignificant, correlation with larvae survival (*r* > 0.05) (Fig. S4B). *Cladosporium* retained three ASVs, and F-ASV252 and F-ASV450 had a positive correlation with larvae survival (*r* < 0, *P* > .05) (Fig. S4B). F-ASV1332 of *Cutaneotrichosporon* exhibited a negative, but nonsignificant, correlation (*r* > 0.05) (Fig. S4B).

To assess whether the enriched strains facilitate larval feeding on plant hosts through functional pathway enrichment, we examined certain positive and negative correlations between numerous pathways identified in bacteria Module 25 and larval growth and development. Several pathways, including LPSSYN-PWY (the super pathway of lipopolysaccharide biosynthesis), PWY-6572 (chondroitin sulfate degradation I), PWY-5392 (reductive TCA cycle II), PWY-3801 (sucrose degradation II sucrose synthase), and PWY-6731 (starch degradation III), were significantly enriched in strains negatively associated with larval survival (Fig. S4C). Metabolic pathways including AEROBACTINSYN-PWY (related to aerobactin biosynthesis), ARGDEG-PWY (related to the super pathway of L-arginine, putrescine, and 4-aminobutanoate degradation), ECASYN-PWY (related to enterobacterial common antigen biosynthesis), ORNARGDEG-PWY (related to the super pathway of L-arginine and L-ornithine degradation), and THREOCAT-PWY (related to the super pathway of L-threonine metabolism) exhibited significant enrichment in bacterial strains that showed a positive correlation with the number of surviving larvae (Fig. S4C). Further analysis of the relationship between the top 10 pathways and larval survival revealed that PWY-6572 (chondroitin sulfate degradation I) and PWY-5392 (reductive TCA cycle II) were associated with co-larval degradation of plant secondary metabolites (*r* < 0, *P* < .05) (Fig. S4E).

In fungi, PWY-7196 (superpathway of pyrimidine ribonucleosides salvage), HSERMETANA-PWY (L-methionine biosynthesis III), and PWY66–409 (superpathway of purine nucleotide salvage) were primarily involved. TYRFUMCAT-PWY (L-tyrosine degradation I) and PWY-7210 (pyrimidine deoxyribonucleotides biosynthesis from CTP) were more abundant in fungal strains that were negatively correlated with the survival rate of larvae (Fig. S4D). GLUCONEO-PWY (Gluconeogenesis I), P221-PWY (Octane Oxidation), PWY-5994 [Palmitate Biosynthesis I (Fungi)], PWY-7235 [Super pathway of Ubiquinol-6 Biosynthesis (Eukaryotic)], and Assimilatory SO4ASSIM-PWY [Sulfate Reduction I (Atory)] pathways were particularly enriched in fungal strains that showed a positive correlation with the number of larvae that survived (Fig. S4D). Further analysis of the relationship between the top 10 pathways and larval survival revealed that only PWY-7210, which involves the biosynthesis of pyrimidine deoxyribonucleotides from CTP, may be involved in the synergistic degradation of plant secondary metabolites (*r* < 0, *P* < .05) (Fig. S4F).

### Metagenomic analysis suggests that the gut microbiome of Zhejiang *H. cunea* can help larvae adapt to new hosts

**Figure 3:**
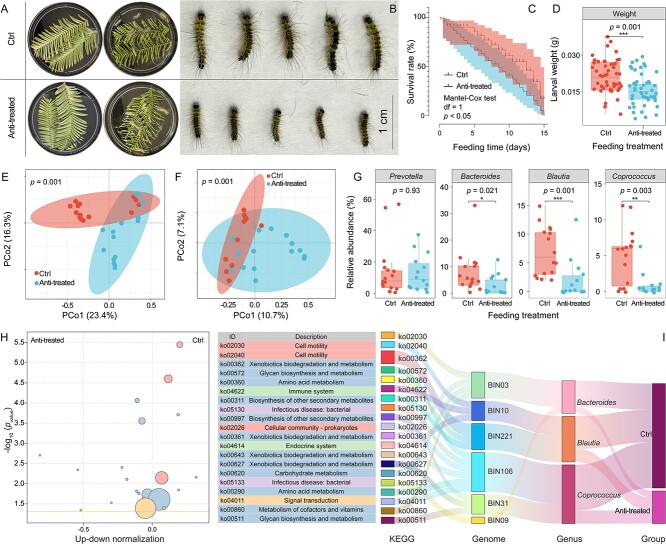
The relationship between gut microbial diversity and performance in Zhejiang *H. cunea* larvae when feeding on unsuitable hosts; (A) feeding *M. glyptostrodoides* to fourth instar *H. cunea* larvae from the Zhejiang population; control group (Ctrl) received water, whereas antibiotics group (antitreated) was administered with antibiotics; (B) comparative analysis of larval morphology in both groups after 15 days; (C) survival rate comparison between the two groups; (D) comparison of larval weight between the two groups; (E) gut bacteria PCoA analysis in both larval groups; (F) Gut fungi PCoA analysis in the two larval groups; (G) comparing relative abundance of target bacterial genera in the gut of the two groups; (H) functional enrichment analysis of gut microbiome differences based on metagenomic data; (I) key function attribution analysis based on metagenomic data.

The antibiotic treatment (antitreated) group had a significantly lower larval survival rate and weight compared to the control (Ctrl) group (*P* < .05) (Fig. 3A–D). Microbial diversity was analyzed, and significant differences occurred between the antitreated and control groups (*P* < .05) (Fig. 3E and F). These results suggest that antibiotic treatment affected the gut microbial structure of *H. cunea* larvae adapted to *M. glyptostroboides* and influenced the compatibility of Zhejiang *H. cunea* larvae with *M. glyptostroboides*. Metagenomic data were analyzed for the target bacteria using amplified gene sequencing. The antibiotic treatment had minimal impact on *Prevotella* but had significant effects on *Blautia*, *Bacteroides*, and *Coprococcus*. The use of antibiotics significantly suppressed the relative abundance of bacteria in these three genera (Fig. 3G).

To determine whether antibiotic treatment affects gut microbiome function, we analyzed metagenomic data and observed differences in KEGG functions. The results indicate that antibiotic treatment significantly inhibits pathways associated with the degradation of toxic substances, including ko00361 (chlorocyclohexane and chlorobenzene degradation), ko00362 (benzoate degradation), ko00627 (aminobenzoate degradation), and ko00643 (styrene degradation) (Fig. 3H and Table S2). The Sankey diagram results indicate that the top 20 pathways with differential enrichment were also enriched in *Blautia*, *Bacteroides*, and *Coprococcus* (Fig. 3I and Table S2). Chlorocyclohexane and chlorobenzene degradation pathways (ko00361) were found in the *Bacteroides* genome (BIN03), whereas the *Blautia* genome (BIN221) contained a benzoate degradation pathway (ko00362). Both ko00627 and ko00643 were associated with aminobenzoate and styrene degradation, respectively (found in the *Coprococcus* genome, BIN31 and BIN106) (Fig. 3I). This result suggests that these three bacterial genera may contribute to the increased tolerance to *M. glyptostroboides* toxicity in the Zhejiang *H. cunea* larvae.

### Transplanting gut microorganisms from Zhejiang *H. cunea* population can help Beijing *H. cunea* larvae adapt to *M. glyptostroboides*

**Figure 4:**
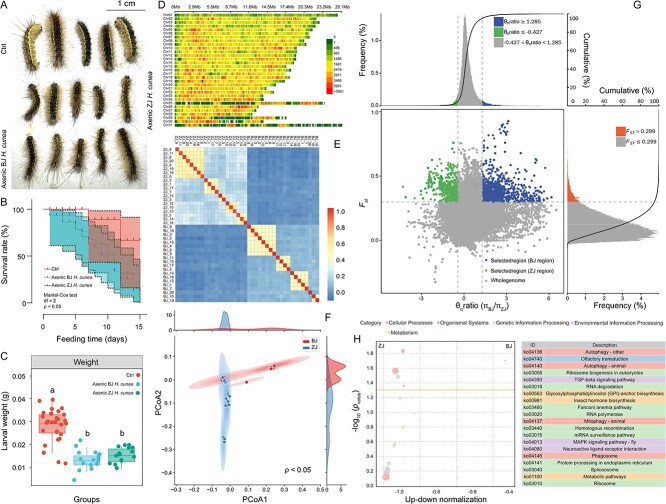
Comparative analysis of the survival rates and weights in axenic Zhejiang and Beijing *H. cunea* larvae, along with genome-wide SNP variation analysis for both groups; (A) morphology of four-instar axenic larvae from both populations after 15 days on Metasequoia diet, compared with non-axenic Zhejiang *H. cunea* larvae as control (Ctrl); (B) survival rate comparison among non-axenic Zhejiang larvae, and axenic larvae from both Zhejiang and Beijing; (C) comparison of body weight among the three groups; (D) chromosomal single nucleotide polymorphisms (SNPs) distribution in *H. cunea*; (E) genome relationship matrix analysis between Zhejiang and Beijing *H. cunea* larvae; Squares closer to the value of 1 indicate larger G values and closer relationships, whereas squares closer to the value of 0 represent smaller G values and more distant relationships ; (F) PCoA analysis based on individual genomic SNP differences; (G) distribution of *θπ* (*θπ* Beijing /*θπ* Zhejiang) and *Fst* values of the genome; the *x*-axis represents the ratio of θπ of Beijing *H. cunea* to *θπ* Zhejiang *H. cunea*, whereas the *y*-axis represents the *Fst* value; gray areas indicate non-selectively cleared regions, whereas blue, green, and orange areas represent selectively cleared regions; (H) functional enrichment analysis of SNP variations in the larval genomes.

To confirm whether the adaptability differences between Zhejiang *H. cunea* and Beijing *H. cunea* are related to gut microbiota, we first compared the survival rates of axenic larvae from the two populations after feeding on *M. glyptostroboides* (Fig. 4A). The methods of plating experiment and bacterial-specific primer PCR confirmed that no bacteria were detected in the axenic *H. cunea* gut (Fig. S5). There was no significant difference in survival (Fig. 4B) or weight (Fig. 4C) between axenic Zhejiang and Beijing *H. cunea* larvae. Resequencing of the genomes of the Beijing and Zhejiang *H. cunea* populations showed that the SNP variation in Zhejiang *H. cunea* population had a relatively high density of SNP variation sites in all 31 of the chromosomes, except Chromosome 1 (Fig. 4D). Genomic relationship matrix analysis, employing SNP markers to estimate inter-individual affinity, revealed population genetic variation between the Beijing and Zhejiang *H. cunea* populations, with a similarity index below 0.4 (Fig. 4E). After dimensionality reduction of re-sequenced SNP via principal component analysis, the Beijing and Zhejiang *H. cunea* populations were distinctly and significantly clustered, with several individuals showing substantial genetic differences (Fig. 4F). In the Zhejiang *H. cunea* population, a smaller portion of the genome (0.8%, 805 windows) exhibited strong selective sweep signals compared to the Beijing *H. cunea* population (1.1% of the genome, 1127 windows) (Fig. 4G). Through gene location comparison, we obtained the corresponding cDNA sequence for windows specifically enriched in the Zhejiang *H. cunea* population and performed KEGG annotation. The results indicated that the Zhejiang *H. cunea* population experienced enrichment in certain functions: ko04136: Autophagy—other; ko04740: Olfactory transduction; ko04140: Autophagy—animal; ko03008: Ribosome biogenesis in eukaryotes; ko04350: Transforming growth factor (TGF)-beta signaling pathway (Fig. 4H and Table S3). None of the enriched functional pathways were previously reported to be associated with tolerance to plant hosts (Fig. 4H and Table S3).

**Figure 5:**
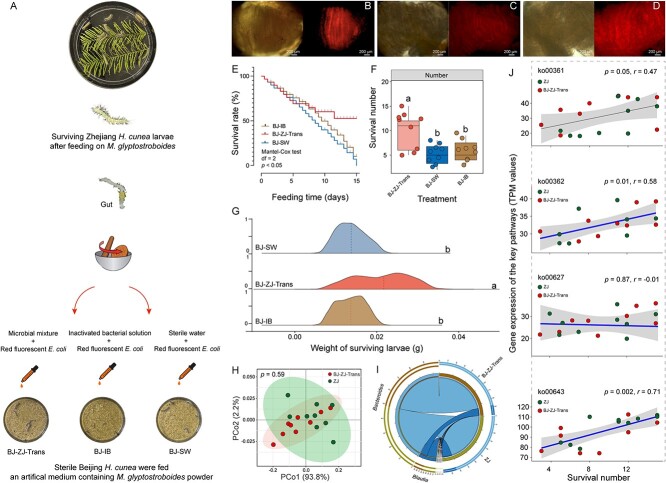
Effects of transferring gut microbiome from Zhejiang *H. cunea* larvae to axenic Beijing *H. cunea* larvae on the latter’s performance on *M. glyptostrodoides*; (A) experimental design: “BJ-ZJ-Trans” denotes axenic Beijing larvae fed an artificial diet infused with gut microorganisms from Zhejiang *H. cunea* larvae; “BJ-SW” refers to axenic Beijing *H. cunea* larvae given an artificial diet with equivalent sterile water; “BJ-IB” involves an artificial diet with an inactivated microbial mixture from Zhejiang *H. cunea* larval guts; (B, C，D) images of gut fluorescence in BJ-ZJ-Trans larvae, BJ-BI larvae, and BJ-SW larvae; (E) comparative analysis of survival rates in axenic Beijing *H. cunea* larvae following various treatments; (F) survival numbers of axenic Beijing *H. cunea* larvae posttreatment; (G) weight comparison of axenic Beijing *H. cunea* larvae across three groups; (H) gut bacterial PCoA of BJ-ZJ-Trans and ZJ larvae following *M. glyptostrodoides* feeding; (I) chord diagram showing species composition between BJ-ZJ-Trans and ZJ larvae; (J) correlation analysis between relative gene expression in the key pathways for plant toxin degradation and the larval survival in BJ larvae (BJ-ZJ-Trans and BJ-SW).

To further illustrate the impact of gut microorganisms on the ability of *H. cunea* to adapt to new hosts, we transplanted gut microorganisms from the Zhejiang *H. cunea* population into the sterile line of the Beijing *H. cunea* population (Fig. 5A). After 15 days of detection, the gut microbiome of Beijing *H. cunea* population gradually expressed stably (Fig. S6). Fluorescence microscopy revealed red fluorescence in the midgut samples of the BJ-ZJ-Trans, BJ-IB, and BJ-SW groups, indicating the successful ingestion of the transferred gut bacteria or inactivated bacteria (Fig. 5B–D). After collecting gut microbiomes from the Zhejiang *H. cunea* larvae, we observed a significant increase in the survival rate (Fig. 5E), survival number (Fig. 5F), and survival larval weight (Fig. 5G) of the Beijing *H. cunea* larvae when fed an artificial diet containing metasequoia powder, compared to the control groups that received either inactivated gut microbiota from Zhejiang *H. cunea* or equivalent sterile water (*P* < .05). Further comparison of the gut microbiota between Zhejiang *H. cunea* larvae (ZJ) and the sterile Beijing *H. cunea* larvae after the transfer of microflora (BJ-ZJ-Trans) showed little difference in species composition and relative abundance of target bacteria genera between the two groups (Fig. 5H and I). Correlation analysis showed a positive correlation between the expression levels of genes (ko00361, ko00362, and ko00643) and the number of larvae that survived (Fig. 5J). However, ko00627 did not exhibit any correlation with the number of surviving larvae in the experiment (Fig. 5J).

## Discussion

*H. cunea* is a highly successful invasive species in China. It has extended its range from northern China to southern subtropical regions and demonstrated high environmental adaptability [26]. As *H. cunea* continues its southward expansion, its plant host range continues to broaden [26]. The first report of this host plant expansion involved its detrimental impact, in 2019, on the gymnosperm *M. glyptostroboides* in Shanghai [27]. We previously found no difference in oviposition preference in the Zhejiang *H. cunea* population between *M. glyptostroboides* and *P. simonii*. The latter is a common host plant for the Beijing *H. cunea* population in northern China. This suggests that the Zhejiang *H. cunea* population has begun adaptation to *M. glyptostroboides*, even though only a small fraction of individuals can complete their life cycle independently [26]. Once a population that is entirely acclimatized to *M. glyptostroboides* emerges, it is anticipated that *H. cunea* will quickly occupy the majority of the *M. glyptostroboides* distribution area due to the reproductive capacity of females that can produce over 900 eggs during a single oviposition event [6, 26]. In the present study, we observed significant compatibility differences between *H. cunea* and major tree species in the southern subtropical regions (Fig. 1, Fig. S1). *H. cunea* exhibited strong adaptability toward mulberry, whereas displaying moderate compatibility toward *C. illinoinensis*, *C. cathayensis*, and *P. simonii*. In contrast, *M. glyptostroboides* showed the lowest compatibility (Fig. 1), suggesting that the adaptation to this species remains incomplete.

Microorganisms are frequently exploited by insects [14, 22, 43-45]. The analysis of *H. cunea* larvae gut microbiome structure following the consumption of various plant hosts revealed significant variations in microbial diversity (Fig. 2 and Figs S2–S4). *M. glyptostroboides* had the most substantial impact on gut bacterial and fungal microbial diversity (Fig. 2 and Figs S2–S4). However, the effects of *C. illinoinensis*, *C. cathayensis*, and *P. simonii* on the alpha diversity of *H. cunea* gut microbiota were comparable (Fig. S2). For beta diversity, the consumption of different foods had a significant impact on the bacterial community structure (Fig. S3). This phenomenon has been reported in many other *Lepidoptera* species as the host plant is a primary influencing factor on the gut microbiota structure [46, 47]. Hannula *et al*. discovered that the gut microbiota of *Mamestra brassicae* larvae are influenced not by the microbiome of its host plant but directly by the plant associated soil microbiome [48]. Given that *M. glyptostroboides* is the most toxic of the five plants to *H. cunea* (resulting in the highest mortality rate), the defensive compounds may be the primary driving factor shaping the larval gut microbiota. For the five plants assessed in this study, differences exist within the plants themselves (in terms of foliar-associated microbiota, nutritional contents, and plant defense substances) and variations also occur in the plant-associated soil microbiota. Determining which specific differences or combinations lead to alterations in the microbiota structure deserves further study. Analysis of bacterial modules indicated a heightened connectivity within the gut microbiota of *H. cunea* larvae (Fig. 2A and E), and specific modules showed a significant negative correlation with the larval survival rate. The samples from the *M. glyptostroboides* group were distributed around the fitting line (Fig. 2B–D and F–H), implying that the prevalence of this module in the *H. cunea* larva gut whereas consuming *M. glyptostroboides* could aid in adaptation to the toxic secondary plant compounds. Analysis of ASVs and their functions enriched in the two modules provided additional evidence that the gut microbiome possesses the ability to enhance *H. cunea* tolerance of *M. glyptostroboides*. This was indicated by a significant enrichment of strains and functions associated with the degradation of toxic plant substances (Fig. S4).

Various gut microorganisms can facilitate insects in tolerating plant toxins [14, 49, 50], including alkaloids, saponins, glucosinolates, and oxalates [14, 49-53]. For instance, the gut microbiome of *Megacopta punctatissima* contains a microbial gene capable of detoxifying oxalate [54]. Similar results have been reported in the bark beetle *Dendroctonus valens* [55]. The leaves of the gymnosperm *M. glyptostroboides* have a thick, hard cuticle and contain a significant number of tannins, phenols, flavonoids, and terpenoids [42, 56, 57], making them a challenging food resource for herbivorous insects. The antibiotic treatment applied to the southern-expanding Zhejiang *H. cunea*, coupled with its feeding on *M. glyptostroboides*, confirmed the role of gut microbiota in facilitating *H. cunea* adaptation to the toxic plant (Fig. 3A–D). Nevertheless, the antibiotic treatment could potentially induce alterations in the leaf microbiota, which might contribute to *H. cunea* mortality. Metagenomic analysis revealed a significant reduction in the predominant gut bacteria (*Bacteroides*, *Blautia*, and *Coprococcus*) following the administration of antibiotics to *H. cunea* (Fig. 3G). These bacteria have been associated with the potential to degrade plant defensive chemicals [58-60]. The subsequent transplantation of gut microbiota from Southern Zhejiang *H. cunea* larvae into Northern Beijing *H. cunea* larvae boosted the survival rate of the latter, which were previously susceptible to *M. glyptostroboides* (Fig. 5). We found that feeding deactivated bacteria from Zhejiang *H. cunea* larvae to antibiotic-treated Beijing *H. cunea* larvae did not enhance their survival rate. This preliminary observation partially ruled out the possibility of the reintroduced bacteria in the treatment group serving as a nutritional substance to improve larval survival rates. In addition, the microbial composition analysis revealed that these strains were found in higher abundance in the gut of the transplanted Beijing *H. cunea* larvae. Collectively, these findings suggest that *Bacteroides*, *Blautia*, and *Coprococcus* potentially contribute to the adaptation of the *H. cunea* pioneer population to *M. glyptostroboides*. Their contribution is likely through detoxification*.* Further isolation of bacteria within these genera and the validation of their detoxification functions will be the focus of future research, serving as a key step to substantiate our hypothesis.

The genome resequencing results of Zhejiang *H. cunea* population revealed a relatively high density of SNP variation sites in all of the chromosomes except for Chromosome 1 (Fig. 4A), and a significant level of population genetic variation was observed between the Beijing and Zhejiang *H. cunea* populations (Fig. 4B). Although the genome resequencing results suggested that the Zhejiang population has undergone a degree of differentiation from the Beijing population, KEGG functional annotation did not indicate a significant enrichment in the Zhejiang *H. cunea* population related to plant host resistance tolerance (Fig. 4D and E). This suggests that the recently established *H. cunea* population in subtropical China has not yet advanced to the evolutionary stage of using host plants that are stable and adapted to subtropical conditions. In this scenario, utilizing microbial partners to enhance its own adaptability appears to be the only choice for *H. cunea*. Some studies have indicated that specific bacterial strains, either individually or in combination, facilitate plant-feeding insects to tolerate plant toxins [14, 49-53]. Nevertheless, the majority of these insects are specialists, feeding on a narrow range of plants [43, 45]. For example, *Acinetobacter* in *Curculio chinensis* specializes in degrading the anti-insect substance tea saponin [43, 45]. *Enterobacteriaceae* sp*.* in *Hylobius abietis* and *Pseudomonas fulva* in *Hypothenemus hampei* have also been documented to assist host insects in overcoming plant defenses [12, 14, 61, 62]. As a polyphagous species of *Lepidoptera*, the coordination of microbiomes in the *H. cunea* gut may be more intricate when it comes to detoxification functions [49, 63]. To confirm whether the gut microbiome of *H. cunea* pioneer populations facilitate their adaptation to new hosts, such as *M. glyptostroboides*, gut microorganisms from Zhejiang *H. cunea* larvae were directly transplanted into the sterile guts of the Beijing population. The increased survival of Beijing *H. cunea* larvae on *M. glyptostroboides* suggests that the gut microbiome helps the larvae to tolerate the defenses of the previously unsuitable plant. The involvement of the gut microbiome may enable the *H. cunea* pioneer population to survive on new hosts, establishing a foundational population to colonize the subtropical zone. This possibility requires further empirical evidence.

Utilizing Beijing *H. cunea* larvae as a reference (representative of the northern *H. cunea* population), we initially assessed the compatibility of Zhejiang *H. cunea* larvae, a pioneer population that moved from the north to the southern regions of China, with a new set of potential subtropical host plants. We demonstrated that the structure and functionality of the gut microbiome in Zhejiang *H. cunea* larvae can be significantly altered by their diet. Genome sequencing did not reveal notable distinctions in host adaptation functions between the two populations. Transplanting gut microbiota from Zhejiang *H. cunea* larvae into Beijing *H. cunea* larvae enhanced their capability to acclimate to an initially unsuitable host plant. This study provides empirical evidence that the gut microbiota of invasive pests, sourced from pioneer populations, can increase pest compatibility with new hosts. This increases their likelihood of successful adaptation to novel habitats.

## Supplementary Material

Supplementary_information_wrae031

## Data Availability

Demultiplexed sequence data are available in the BIG Submission (PRJCA022940: CRA014601) and the NCBI databases (Bio-Project ID: PRJNA1024085).
