# Supplementary material for: Gut microbiota facilitate adaptation of invasive moths to new host plants
Source: ISME J. 2024 Feb 29;18(1):wrae031. doi: 10.1093/ismejo/wrae031 (PMC10980833; doi:10.1093/ismejo/wrae031)
Supplement: Supplementary_information_wrae031 [file supplementary_information_wrae031.docx]

**Supplementary information**

**Gut microbiota facilitate adaptation of invasive moths to new host plants**

**Shouke Zhang^1,†,*^, Feng Song^1,†^, Jie Wang^1,†^, Xiayu Li^2^, Yuxin Zhang^2^, Wenwu Zhou^3,*^, Letian Xu^2,*^**

^1^State Key Laboratory of Subtropical Silviculture, Zhejiang A&F University, Hangzhou, P. R. China.

^2^State Key Laboratory of Biocatalysis and Enzyme Engineering, School of Life Sciences, Hubei University, Wuhan, P. R. China

^3^Ministry of Agricultural and Rural Affairs Key Laboratory of Molecular Biology of Crop Pathogens and Insect Pests & Key Laboratory of Biology of Crop Pathogens and Insects of Zhejiang Province, Institute of Insect Science, Zhejiang University, Hangzhou, P. R. China


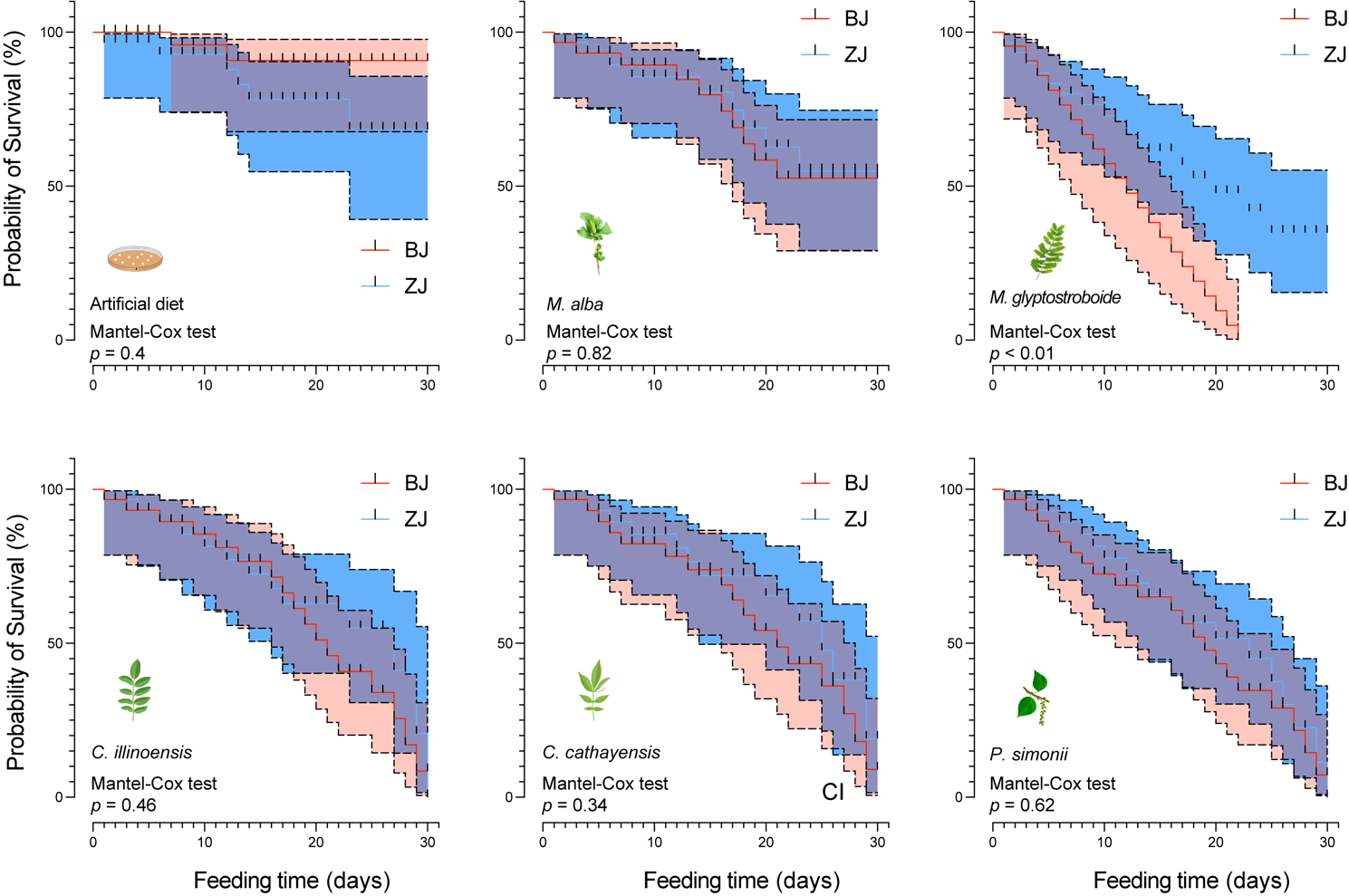


**Figure S1** Comparison of survival rates of Beijing and Zhejiang *H. cunea* larvae after being fed different diets


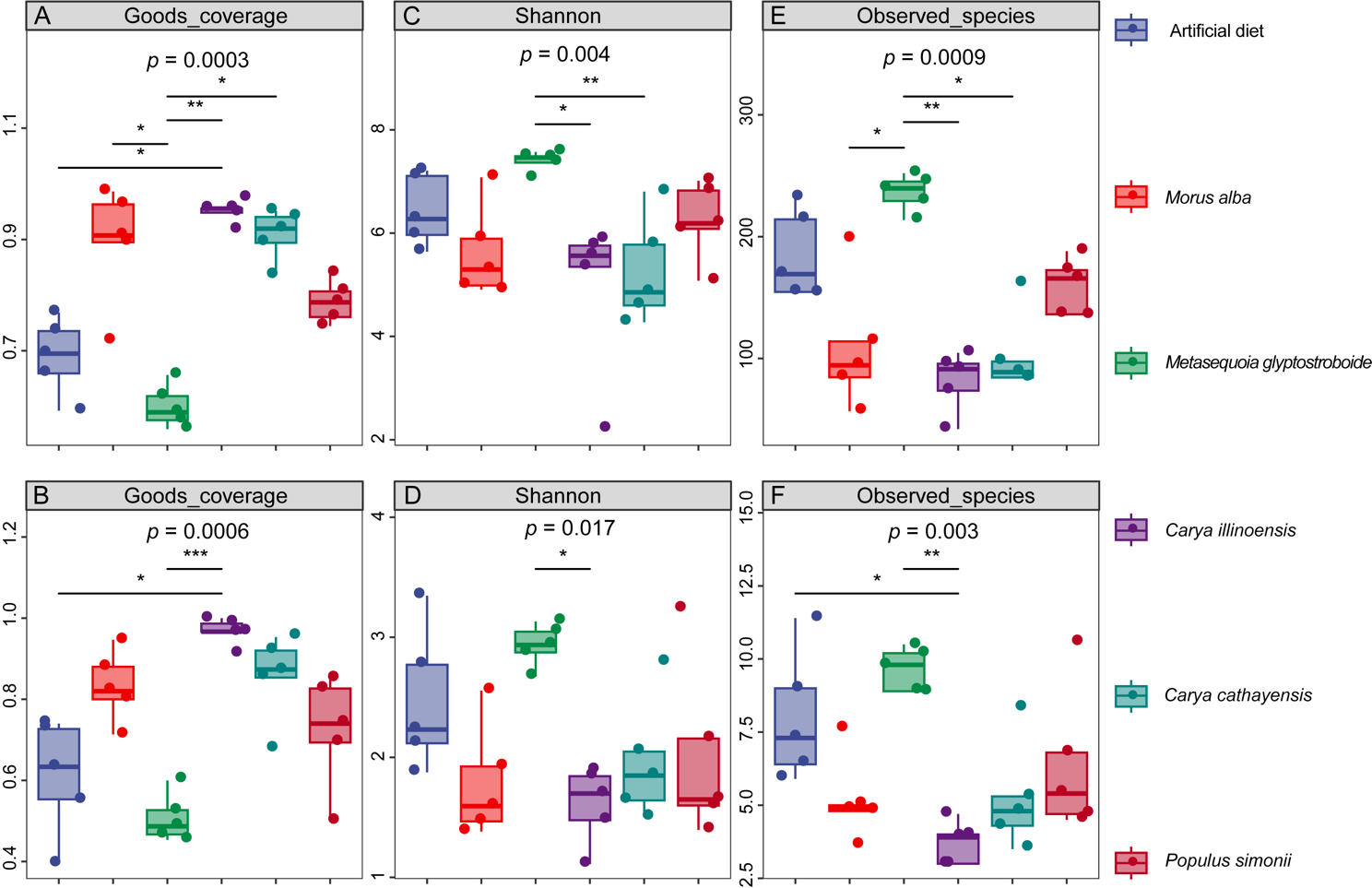


**Figure S2** Analyzing the differences in *H. cunea* gut microbiome alpha diversity after feeding on different diets. The up row shows the alpha diversity index for bacteria, including the Goods_coverage index (A), the Shannon index (C), and the Observed_species (E). The second row is for fungi, including the Goods_coverage index (B), the Shannon index (D), and the Observed_species (F). The levels of significance are indicated as 0.001***, 0.01**, and 0.05*.


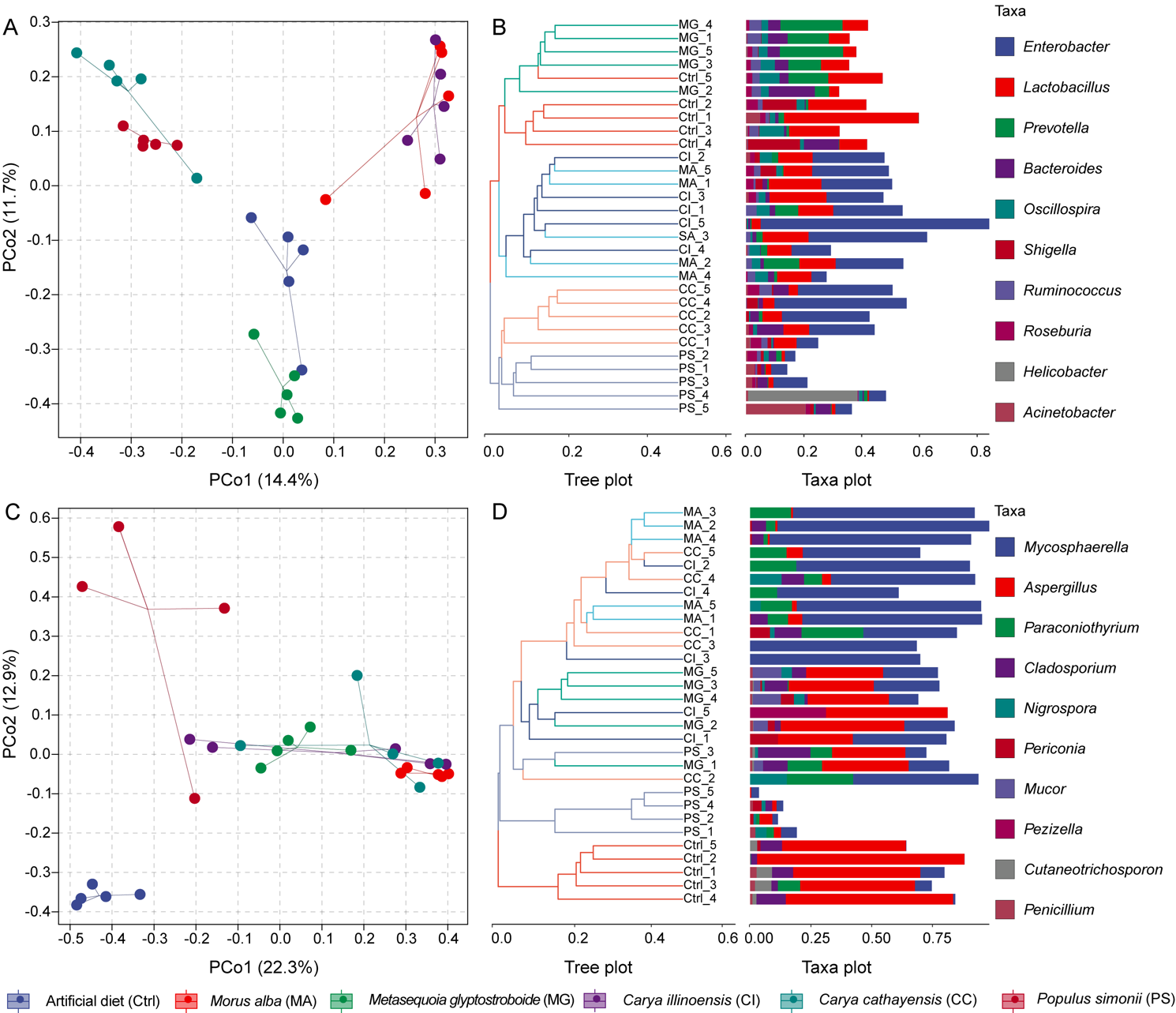


**Figure S3** Effects of different host diets on the beta diversity of the gut microbiome of *H. cunea* larvae. (A) Principal coordinate analysis was based on the 16S rRNA gene weighted Bray–Curtis distances of these six groups, respectively and used ANOSIM of variance. The six colors represent different sample sources (CK, SA, SH, M, L, and Y). CK: Artificial feed; SA: *Morus alba*; SH: *Metasequoia glyptostroboide*; M: *Carya illinoensis*; L: *Carya cathayensis*; Y: *Populus simonii*. (B) Microbial composition of bacteria shown at the genus level. Shown are changes in the relative abundances of the 10 most abundant species in CK, SA, SH, M, L, and Y samples. (C) Principal coordinate analysis of fungi was based on ITS gene weighted Bray–Curtis distances of these six groups, respectively. (D) Microbial composition of fungi shown at the genus level.


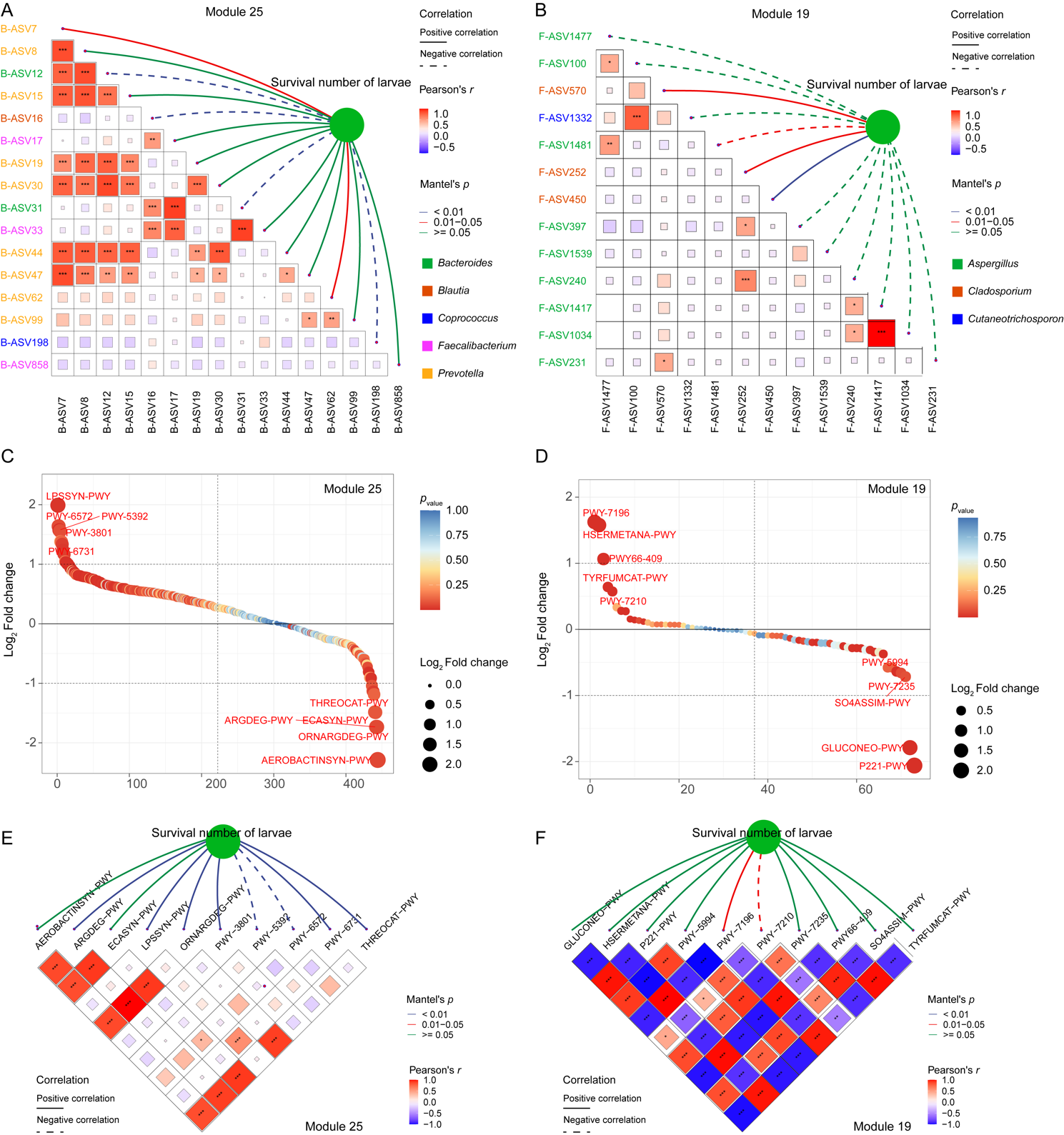


**Figure S4** Relationship between specific enrichment of ASV in bacterial Module 25 and fungal Module 19 and the survival of larvae. (A) Correlations between the bacterial ASVs in Module 25 with the survival number of larvae. The colors of ASVs represent different genera of bacteria. The *p* values were showed by the color of the connected lines. Positive correlations were connected using solid lines, and negative correlations were connected using dashed lines. (B) Correlations between fungous ASVs in Module 19 with the survival number of larvae. Differential enrichment function analysis between positively correlated ASVs and negatively correlated ASVs in Module 25 (C) and Module 19 (D). The top10 enriched pathways were marked in red. Correlations between the top 10 pathways from Module 25 (E) and Module 19 (F) with survival number of larvae.

**
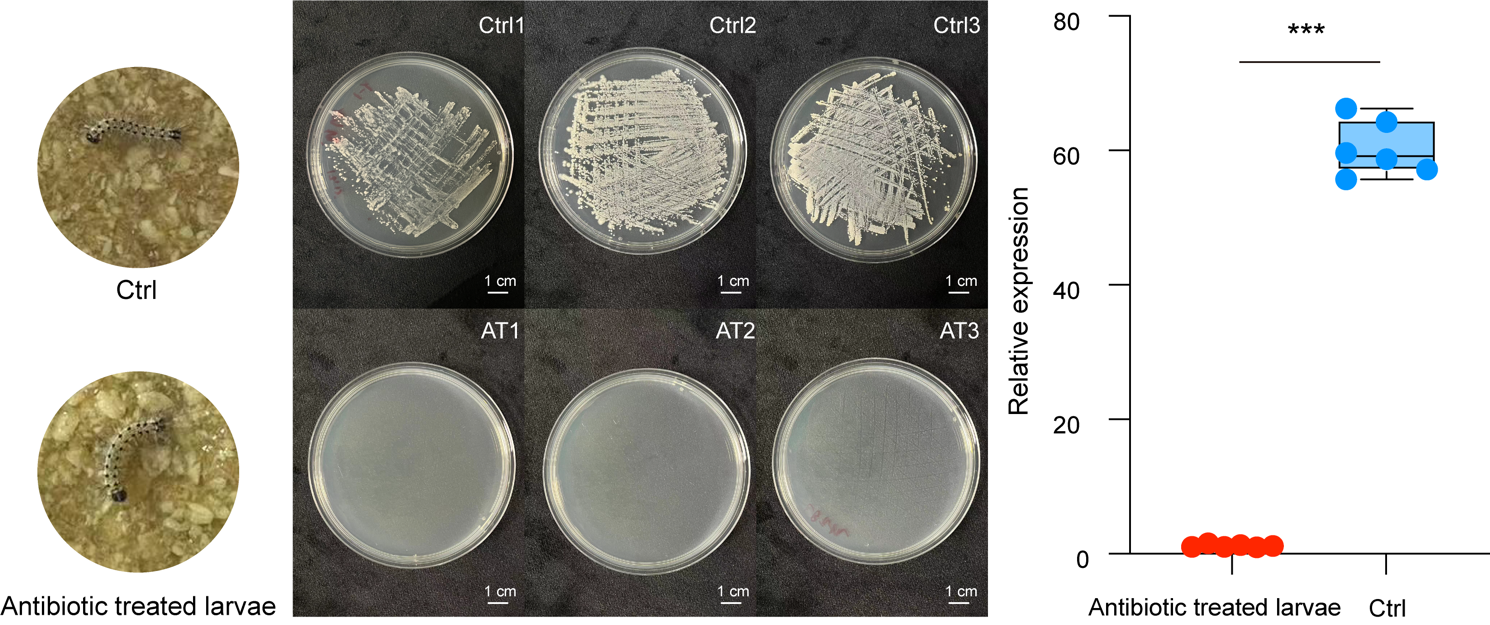
**

**Figure S5** The results of gut microbiome coating of *H. cunea* in antibiotic treated group and untreated group


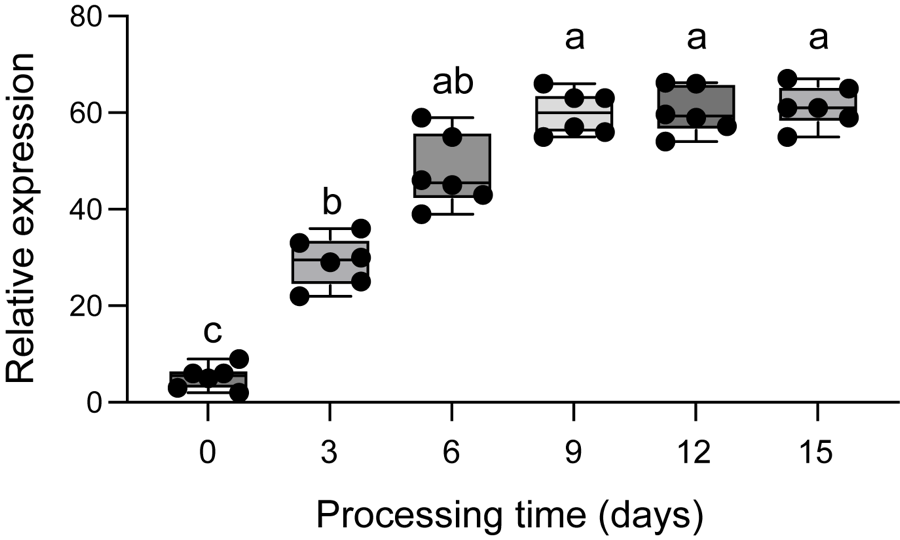


**Figure S6** Expression of intestinal bacteria in Beijing *H. cunea* larvae after the transfer of gut microbiome from Zhejiang *H. cunea* larvae

**Table S1** Permutational multivariate analysis of variance between different host feeding groups

| Group1 | Group2 | Sample size | Bacteria | | Fungus | |
| --- | --- | --- | --- | --- | --- | --- |
|  |  |  | pseudo*-F* | *p*_value_ | pseudo-*F* | *p*_value_ |
| All | - | 30 | 3.11 | 0.00 | 4.36 | 0.00 |
| Artificial diet | *Morus alba* | 10 | 2.63 | 0.01 | 14.03 | 0.01 |
| Artificial diet | *M. glyptostroboide* | 10 | 2.81 | 0.01 | 7.31 | 0.01 |
| Artificial diet | *C. illinoensis* | 10 | 3.37 | 0.01 | 4.83 | 0.01 |
| Artificial diet | *C. cathayensis* | 10 | 3.47 | 0.01 | 6.96 | 0.01 |
| Artificial diet | *P. simonii* | 10 | 2.75 | 0.01 | 6.44 | 0.01 |
| *M. alba* | *M. glyptostroboide* | 10 | 3.52 | 0.01 | 4.80 | 0.01 |
| *M. alba* | *C. illinoensis* | 10 | 1.18 | 0.20 | 1.52 | 0.24 |
| *M. alba* | *C. cathayensis* | 10 | 3.53 | 0.01 | 1.27 | 0.29 |
| *M. alba* | *P. simonii* | 10 | 3.01 | 0.01 | 9.36 | 0.01 |
| *M. glyptostroboide* | *C. illinoensis* | 10 | 3.78 | 0.01 | 1.39 | 0.18 |
| *M. glyptostroboide* | *C. cathayensis* | 10 | 4.09 | 0.01 | 3.00 | 0.01 |
| *M. glyptostroboide* | *P. simonii* | 10 | 3.28 | 0.01 | 4.59 | 0.01 |
| *C. illinoensis* | *C. cathayensis* | 10 | 4.03 | 0.01 | 0.77 | 0.59 |
| *C. illinoensis* | *P. simonii* | 10 | 3.37 | 0.01 | 3.35 | 0.02 |
| *C. cathayensis* | *P. simonii* | 10 | 2.11 | 0.02 | 4.15 | 0.01 |

The levels of significance are indicated as 0.01, and 0.05.

**Table S2** Specific enrichment of synergistic microbiome functional pathways tolerant to *M. glyptostrodoides* toxicity

| ID | Ctrl | AT | log_2_ (Flod change) | *p*_value_ | FDR | KEGG_A_class | KEGG_B_class | Pathway |
| --- | --- | --- | --- | --- | --- | --- | --- | --- |
| ko00290 | 25203 | 22749 | -0.148 | 0.025 | 0.302 | Metabolism | Amino acid metabolism | Valine, leucine and isoleucine biosynthesis |
| ko02060 | 20148 | 22142 | 0.136 | 0.041 | 0.404 | Environmental Information Processing | Membrane transport | Phosphotransferase system (PTS) |
| ko02026 | 15502 | 13461 | -0.204 | 0.007 | 0.148 | Cellular Processes | Cellular community - prokaryotes | Biofilm formation - Escherichia coli |
| ko02030 | 13299 | 10504 | -0.340 | 0.000 | 0.003 | Cellular Processes | Cell motility | Bacterial chemotaxis |
| ko00620 | 13204 | 14114 | 0.096 | 0.019 | 0.258 | Metabolism | Carbohydrate metabolism | Pyruvate metabolism |
| ko02040 | 11150 | 7409 | -0.590 | 0.000 | 0.001 | Cellular Processes | Cell motility | Flagellar assembly |
| ko00360 | 9280 | 10747 | 0.212 | 0.000 | 0.012 | Metabolism | Amino acid metabolism | Phenylalanine metabolism |
| ko00362 | 5180 | 7684 | 0.314 | 0.000 | 0.006 | Metabolism | Xenobiotics biodegradation and metabolism | Benzoate degradation |
| ko00511 | 5114 | 4284 | -0.256 | 0.041 | 0.404 | Metabolism | Glycan biosynthesis and metabolism | Other glycan degradation |
| ko05133 | 4907 | 5522 | 0.171 | 0.024 | 0.302 | Human Diseases | Infectious disease: bacterial | Pertussis |
| ko00860 | 3672 | 4185 | 0.189 | 0.034 | 0.365 | Metabolism | Metabolism of cofactors and vitamins | Porphyrin and chlorophyll metabolism |
| ko00572 | 3106 | 2114 | -0.555 | 0.000 | 0.010 | Metabolism | Glycan biosynthesis and metabolism | Arabinogalactan biosynthesis - Mycobacterium |
| ko00627 | 2294 | 2783 | 0.279 | 0.018 | 0.258 | Metabolism | Xenobiotics biodegradation and metabolism | Aminobenzoate degradation |
| ko00643 | 1886 | 2361 | 0.324 | 0.016 | 0.247 | Metabolism | Xenobiotics biodegradation and metabolism | Styrene degradation |
| ko00361 | 1537 | 2227 | 0.535 | 0.008 | 0.148 | Metabolism | Xenobiotics biodegradation and metabolism | Chlorocyclohexane and chlorobenzene degradation |
| ko04614 | 1285 | 868 | -0.566 | 0.015 | 0.247 | Organismal Systems | Endocrine system | Renin-angiotensin system |
| ko00311 | 671 | 1047 | 0.641 | 0.004 | 0.118 | Metabolism | Biosynthesis of other secondary metabolites | Penicillin and cephalosporin biosynthesis |
| ko00997 | 547 | 281 | -0.963 | 0.007 | 0.148 | Metabolism | Biosynthesis of other secondary metabolites | Biosynthesis of various secondary metabolites - part 3 |
| ko04011 | 247 | 454 | 0.880 | 0.030 | 0.345 | Environmental Information Processing | Signal transduction | MAPK signaling pathway - yeast |
| ko04152 | 231 | 727 | 1.657 | 0.046 | 0.431 | Environmental Information Processing | Signal transduction | AMPK signaling pathway |
| ko05130 | 111 | 371 | 1.745 | 0.005 | 0.118 | Human Diseases | Infectious disease: bacterial | Pathogenic Escherichia coli infection |
| ko04622 | 24 | 239 | 3.306 | 0.002 | 0.068 | Organismal Systems | Immune system | RIG-I-like receptor signaling pathway |

**Table S3** Functional pathways specifically enriched in Zhejiang population genome

| KEGG_A_class | KEGG_B_class | Pathway | Out | All | *p*_value_ | Q_value_ | Pathway ID | Genes | K_IDs |
| --- | --- | --- | --- | --- | --- | --- | --- | --- | --- |
| Cellular Processes | Transport and catabolism | Autophagy - other | 3 | 53 | 0.015 | 0.175 | ko04136 | Hcu12G002430.1; Hcu10G000090.1; Hcu21G001230.1 | K17890+K17908+K08337 |
| Organismal Systems | Sensory system | Olfactory transduction | 1 | 2 | 0.020 | 0.175 | ko04740 | Hcu01G005110.1 | K19695 |
| Cellular Processes | Transport and catabolism | Autophagy - animal | 4 | 118 | 0.027 | 0.175 | ko04140 | Hcu12G002430.1; Hcu10G000090.1; Hcu21G001230.1; Hcu22G002500.1 | K17890+K17908+K08337+K10435 |
| Genetic Information Processing | Translation | Ribosome biogenesis in eukaryotes | 3 | 72 | 0.033 | 0.175 | ko03008 | Hcu30G000290.1; Hcu11G003640.1; Hcu11G003630.1 | K06943+K14552+K14552 |
| Environmental Information Processing | Signal transduction | TGF-beta signaling pathway | 2 | 34 | 0.044 | 0.185 | ko04350 | Hcu30G000170.1; Hcu30G000160.1 | K06766+K06766 |
